# The Role of Interoception in the Pathogenesis and Treatment of Anorexia Nervosa: A Narrative Review

**DOI:** 10.3389/fpsyt.2020.00281

**Published:** 2020-04-17

**Authors:** Aimée Margarita Marisol Catherine Jacquemot, Rebecca Park

**Affiliations:** Medical Sciences Office, John Radcliffe Hospital, Oxford University, Oxford, United Kingdom

**Keywords:** anorexia, anorexia nervosa, interoception, pathogenesis, treatment

## Abstract

Anorexia nervosa (AN) is a psychiatric illness characterized by extreme overvaluation of weight and disturbed eating. Despite having the highest mortality rate of any psychiatric illness, the etiology and neurobiology of AN are poorly understood. A growing body of research has begun to elucidate the role of reward processing, as well as cognitive and limbic networks, in the symptomology of AN. However, these advances have so far failed to contribute therapeutically, suggesting a new understanding may be necessary. A disturbance in the interoceptive system, involved in perceiving and interpreting the physiological condition of the body, has recently been proposed as a central mechanism of pathology in AN, through links to hunger and satiety, risk prediction errors, emotional awareness, and body dysmorphia. This review summarizes the existing literature in order to clarify possible underlying mechanisms and proposes a novel model of the neuro-circuitry of AN. Detailed neuroanatomical studies and new methods for studying interoception may allow further refinement of this model and the development of improved treatment.

## Introduction

Anorexia nervosa (AN) is a psychiatric condition defined by extreme overvaluation of shape and weight and disturbed eating, resulting in clinically significant impairments in health and psychosocial function due to self-starvation (1). The symptoms of AN are compounded by resistance to treatment and severe denial (2), resulting in the highest mortality rate of any psychiatric illness (3). AN is thought to have a complex pathogenesis—genetically-determined trait alterations confer a vulnerability which is exacerbated by premorbid experiences and environmental risk factors (4), with state alterations secondary to malnutrition sustaining or accelerating the illness (2). Nonetheless several hypotheses exist surrounding the neurobiology of AN, supported by a growing body of research (reviewed in Kaye et al. (2). In particular, studies have focused on the dysregulation of monoamine neurotransmitters and reward processing in AN (2), as well as on the imbalance between dorsal (cognitive) and ventral (limbic) networks allowing suppression of intuitive responses to satisfy long-term goals (*e.g.* staying thin) over basic needs (2). While these mechanisms explain much of the symptomology of AN, therapeutic applications have been limited, suggesting new understanding may be necessary. Emergent evidence suggests deficits in interoception may play a role in the pathogenesis of this enigmatic condition.

**Figure 1:**
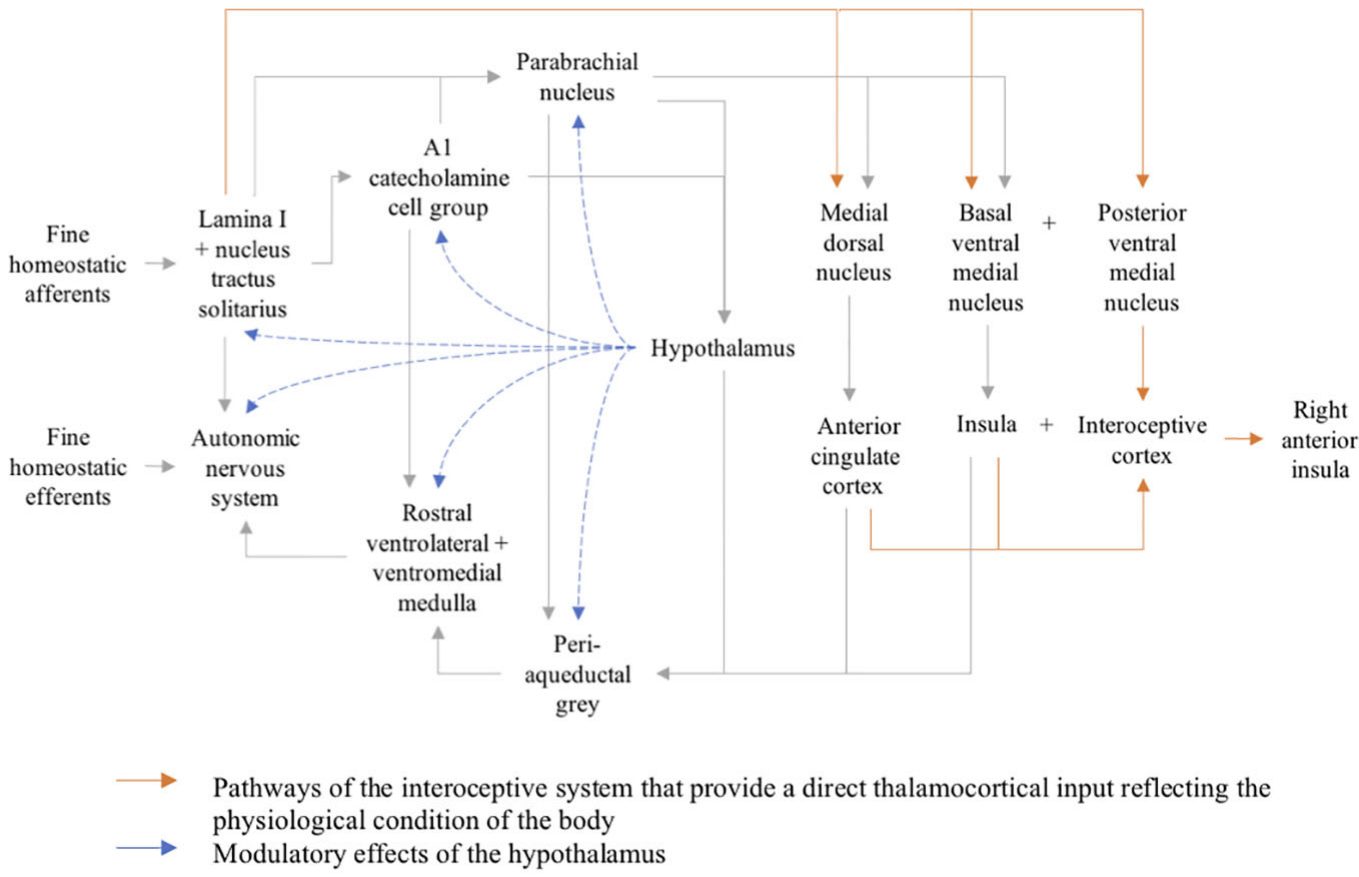
Organizational map of the interoceptive system.

Interoception refers to the perception and integration of afferent signals which represent the homeostatic and physiological condition of the body (5). These involve highly resolved feelings including pain, temperature, itch, muscular and visceral sensations, hunger, and thirst (6). Primary interoceptive afferents are thought to be located in lamina I of the spinal cord, constituting a parallel system to sympathetic and parasympathetic afferents (**Figure 1**) (5). Neuroanatomical and functional neuroimaging studies have suggested a central role for the insular cortex in this system, specifically the anterior insula (AI) (5, 7–9). A recent meta-analysis has elaborated on the role of the insular cortex in the integration of perception, emotions, thoughts, and plans. This appears to occur through four functionally distinct regions in the insula mapping onto sensorimotor, cognitive, social–emotional, and olfactory–gustatory networks in the brain. The anterior dorsal insula demonstrates significant overlap of these domains and has therefore been hypothesized to serve as a central multimodal integration region which generates a coherent experience of the world in which interoception plays a key role (10). Interestingly, the mid-dorsal insula which links somato- and viscerosensory stimuli to the anterior insula has been repeatedly shown to be altered in patients with AN (11). In fact, a strong literature of neuroimaging studies have referred to aberrant insular function in AN, specifically in the context of altered interoception.

Altered interoceptive awareness (IA) has long been suggested as a precipitating and reinforcing factor in AN—early clinical descriptions of AN patients by Hilde Bruch in 1962 underlined “a failure of recognizing bodily states as a characteristic” (12). In addition, IA has constituted an integral part of the Eating Disorder Inventory (EDI) since its conception by Garner et al. in 1983 (13), supported by recent prospective studies which validate its use as a diagnostic criterion (14) and risk factor for the development of disordered eating (15). However, IA considers a single aspect of interoception—terminology remains confused in the literature as studies fail to differentiate interoceptive sensitivity (IS), measured by objective tests of interoceptive accuracy, and interoceptive awareness (IA), gaged using self-report questionnaires such as the EDI. A recent paper demonstrated that deficits in IS and IA were in fact not correlated in patients with AN—IA was instead correlated with assessed levels of depression and anxiety (16). The authors therefore suggested that IA is not a measure of visceral sensitivity itself but rather a reflection of disorder-specific dysfunctional thoughts and feelings impacting the *interpretation* of visceral signals. Nonetheless, there is evidence to suggest that both IS and IA are disrupted in AN. This narrative mini review aims to explore the possible role of interoception in the pathogenesis and treatment of AN through exploration of both the symptomatology and neuroanatomical correlates and thereby suggest a novel model of the neuro-circuitry of anorexia.

## Method

Given the nature of this paper as a narrative mini review, a PubMed literature search was conducted primarily as an overview of the existing literature at the time of writing (2019). MeSH terms included were “anorexia nervosa OR eating disorder OR eating disorders” AND “interoception OR interoceptive”. However, given the mixture of terminology in the literature in this field in particular, including the frequent use of synonyms for interoception, after the initial search had been done bibliographies of relevant articles were used to find any other relevant literature.

## Results

The literature search yielded 259 papers, of which 245 were in English. 229 results remained in searching for only full text articles. 33 were considered most relevant and specific to the contents of this narrative mini review (see **Table 1**).

**Table 1:** Study characteristics of included papers.

References	Method and sample size
Robinson et al. (17)	Visual analog scale ratings of satiety, n = 22
Bluemel et al. (18)	MRI and 13C lactose ureide breath test, self-reported sensations of satiation, n = 60
Coddington et al. (19)	Direct introduction of liquid into the stomach and self-report questionnaires, n = 3
Nakai et al. (20)	Linear visual analog techniques and hunger ratings, n = 17
Duncan et al. (21)	Genome wide association study, n = 14477
Herbert et al. (22)	Heartbeat perception task, intuitive eating scale and BMI, n = 111
Wang et al. (23)	fMRI and BOLD study during dynamic gastric balloon distension, n = 18
Santel et al. (24)	fMRI study in response to visual food and non-food stimuli, n = 23
Wierenga et al. (25)	Delay discounting monetary decision task and fMRI, n = 40
Geeraerts et al. (26)	Gastric barostat study, n = 14, satiety drinking test and visual analog scale ratings, n = 18
Obendorfer et al. (27)	fMRI and anticipation task, n = 28
Wagner et al (28)	fMRI and sucrose/water administration, n = 32
Strigo et al. (29)	fMRI and painful heat stimuli, n = 22
Frank et al. (30)	fMRI and reward conditioning task, n = 63
Peuschoff et al. (31)	fMRI and gambling task with constantly changing risk, n = 19
Damasio et al. (32)	Neurology book on mind/body dualism
Pollatos et al. (33)	Heartbeat perception task (HPT) and EEG during emotional picture presentation, n = 32
Uher et al. (34)	fMRI and visual and complex gustatory food-related stimuli, n = 18
Terasawa et al. (35)	fMRI and questionnaire on emotional awareness, bodily awareness, and personal possessions, n = 18
Taylor et al. (36)	Toronto Alexithymia Scale (TAS) and the Eating Disorder Inventory, n = 312
Bourke et al. (37)	TAS, n = 78
Herbert et al. (38)	HPT, TAS, BDI-2, n =155
Beadle et al. (39)	TAS, Interpersonal Reactivity Index, Minnesota Multiphasic Personality Inventory-2, n = 42
Rezek et al. (40)	Manipulation of perceived control and questionnaires related to eating and body image = 40
Tsakris et al. (41)	HBT and hand illusion task, n = 46
Zucker et al. (42)	Variety of neuropsychological and self-report measures, n = 59
Sachdev et al. (43)	fMRI and images of self and nonself, n = 20
Eshkevari et al. (44)	Self-report measures of ED psychpathology *e.g.* EDI-3 subscales, DASS-21 and self-objectification questionnaire, n = 167
Cavanna et al. (45)	Review of literature focusing on the structure and function of the precuneus
Devue et al. (46)	fMRI, self and nonself images, n = 20
Boswell et al. (47)	Review of literature focusing on interoceptive exposure as a method of treatment
Fairburn et al. (48)	Review of literature focusing on the use of cognitive behavioral therapy for eating disorders
Khalsa et al. (49)	Ongoing randomized control trial using infusions of isoproterenol to investigate the effectiveness of IE as a treatment for AN

### Interoceptive Sensitivity

Objective tests of interoception, otherwise known as measures of interoceptive sensitivity are most commonly heartbeat perception tasks (HPT) in which subjects silently count their heartbeats in a given period of time without taking their pulse or using other forms of manipulation to aid the counting process. Patients with AN generally show a reduction in the ability to accurately perceive their heartbeat compared to controls (16, 50), with no significant improvement over the course of treatment (50). This interoceptive task in an fMRI study was specifically related to greater activity in the right AI in AN patients than healthy controls (11), suggesting problems in interoception occur at the level of the insula rather than primary afferents. Since the insula is known to be at the center of interoception in the brain (5, 7–9), this study also supports the validity of the HPT in detecting processes involved in interoception.

However, HPTs are influenced by attention and motivation as well as subjects’ beliefs and expectancies with respect to their heart rates. It has been suggested that a heartbeat detection method, which involves judging whether heartbeat sensations are simultaneous with external stimuli, suffers from fewer confounds than the tracking method (51) but its use in literature is limited. In addition, while these tasks are often used to represent a definite measure of IS, the generalizability of cardiac awareness to other interoceptive processes is unclear (52) with evidence suggesting that accurate detection of bodily sensations across different sensory modalities is not related (53). Since the literature on IS in AN is dominated by HPTs, it is clear that much scope remains for new methods in future research before application to treatment possibilities is considered.

#### Hunger and Satiety

Hunger and satiety are important examples of interoceptive modalities which are disturbed in AN. Patients are significantly more likely to report satiety in questionnaires and visual analog scale (VAS) ratings following meal consumption, showing not only increased but also prolonged satiety (17, 18) compared to controls. One explanation considers altered interoceptive sensitivity to gastric distension in AN—an early study reported a failure of three individuals with AN to detect small volumes of a liquid milkshake delivered directly into the stomach (19). However, the sample size hinders valid conclusions being drawn from this data, and the invasive nature of the procedure has led to few attempts to replicate these results. Moreover, a recent study using MRI to measure postprandial gastric volumes found that one in three AN patients reported fullness and no hunger even when the stomach is completely empty (18). This implies that satiety disturbances in AN may be due to the interpretation and perception of visceral signals (IA) rather than actual differences in visceral sensitivity. Alternatively, modalities of interoception other than gastric distension may be involved—hunger ratings in AN patients have been shown to paradoxically decrease in response to insulin induced hypoglycaemia (20). In addition, recent genome wide association studies (GWAS) have identified AN-associated loci with correlations to specific metabolic phenotypes (21). While it is still unclear whether these are predisposing genetic risk factors or state-related changes in gene activation, metabolism is a well-established component of interoception and so abnormal phenotypes may contribute to faulty IA in AN. Irrespective of the affected interoceptive modality, it is unsurprising that problems with perceiving hunger and satiety can result in dysfunctional eating habits, with recent research suggesting this is due to a departure from intuitive eating which would usually serve to maintain a normal weight (22). fMRI studies have shown that self-reports of satiety and fullness are correlated with insular responses (23), lending support to the insula and its role in interoception as a central candidate for the symptoms of AN.

However, it is important to note that a number of factors other than faulty interoception may contribute to problems with hunger and satiety perception in AN. One explanation points to the role of attentional mechanisms in visual areas during hunger and decreased food-related somatosensory processing *e.g.* gustatory perception/imagination of taste in satiety due to weaker inferior parietal activation (24). Indeed, a recent study using a delay discounting monetary decision task found a failure to increase activation of reward circuitry when hungry compounded by an elevated response in cognitive control circuitry independent of metabolic state (25). This strongly supports a role for faulty reward processing and overactive cognitive circuits focused on long term goals in suppressing hunger and promoting satiety. Another explanation could be that higher satiety ratings and reduced reporting of hunger may represent a “secondary gain” in AN patients undergoing weight rehabilitation—attempts by patients to excuse eating less. Anxiety, often comorbid with AN can also influence results through reducing gastric accommodation (26). Nonetheless it is logical that misinterpretation of hunger/satiety signals could generate food avoidance and disturbed eating habits in AN.

#### Risk Prediction Errors

Dysregulated interoception may also contribute to food avoidance through generation of risk prediction errors in the body; abnormal mapping of interoceptive signals in AN results in erroneous judgements about the internal state of the body, therefore causing a mismatch between expected and actual outcomes. This produces an interoceptive prediction error and negative affective state which may precipitate or propagate abnormal eating behaviors. This mechanism is supported by an emerging neuroimaging literature—on exposure to food or its conditioned stimuli, weight restored AN patients exhibit increased insular and cingulate cortex activation as well as intensified interoceptive sensations, relative to healthy controls (27) yet have a decreased insular response to the taste of food stimuli (28). The exaggerated anticipatory neural response but correspondingly dampened reaction to stimulation may represent a deficit in central integration of expected *versus* experienced interoception in the AI. These results have also been replicated with painful thermal skin stimulation (29). In addition, recent research has shown that patients with AN show increased orbitofrontal cortex activation when receiving reward unexpectedly (30), lending further support to an interoceptive mechanism as the OFC has been explicitly linked to risk prediction errors (31).

#### Emotional Awareness

Since the early 90s, interoception and the physiological state of the body have been thought to play a role in the subjective experience of emotion (32), with recent findings demonstrating that interoceptive sensitivity is positively correlated with the experienced intensity of feelings (33). In addition, internal motivational factors such as hunger and satiety have been shown to modulate the processing of gustatory food related stimuli in the insula (34), with the right anterior insula being specifically activated by both interoceptive and emotional tasks, suggesting these are functionally associated and may underpin subjective experience of the emotional state (35). In AN, faulty interoception may therefore explain difficulties with emotion processing, including high rates of alexithymia characterized by a reduced ability to identify and describe one’s feelings, difficulty in distinguishing feelings from the bodily sensations of emotional arousal, and a tendency to focus on external rather than internal events (36). Specifically, rates of alexithymia are almost 77% in AN patients compared with 6.7% in control subjects (37), with IA inversely associated with all features of alexithymia (38). This appears to not be solely state-related as both recovered and currently ill subjects have greater rates of alexithymia, although the difference is exaggerated in currently ill subjects due to high rates of depression (39).

Patients with AN also demonstrate a lowered ability to self-regulate emotions including poor reappraisal (reframing thoughts and emotions in a more positive direction) and high suppression (modifying one’s behavioral response to an event) (39). Intense negative affective reactions and sensations of lack of control may therefore be controlled using psychologically unhealthy strategies—strict food restriction and refocusing the attention on weight or figure has been shown to be increased in subjects experiencing experimentally induced low control than those in a high control situation (40). This supports an alternative mechanism by which abnormal interoception may contribute to AN eating behaviors.

#### Body Dysmorphia

Interoceptive awareness has also been linked to body dysmorphia which predictably maintains negative eating behaviors. When interoception is impaired, subjects cannot rely on internal signals to perceive physical changes that accompany weight loss, leading to perpetual body image dissatisfaction and an increased tendency to self-objectify due to increased reliance on exteroceptive signals. Much of the research on body image disturbance in AN has examined cognitive components such as body dissatisfaction and perceptual aspects including visual image distortion but has neglected the subjective *experience* of the body. For example, IS can predict the malleability of body representations—a study found that healthy subjects with low scores on an HBT experienced a stronger illusion of ownership in the right-hand illusion task, despite no apparent proprioceptive deficits (41). In AN, poor IS is associated with an over-evaluation of self-image despite normal perception of others (42, 43), which is also seen in recovered patients suggesting this may be a trait which enables the development of AN rather than a state-related change (44). Recent evidence has emphasized the role of the precuneus in mental self-representations (45) with AN patients showing deactivation of the precuneus when viewing self-images (43), as well as across a number of interoceptive modalities (11). Similarly to studies in the precuneus, evidence also demonstrates a link between the anterior insula and self-recognition (46). This implies the possibility of parallel roles for the AI and the precuneus in linking interoceptive and contextual information to influence emotional experience (35).

### Treatment Implications

Despite recent attention towards interoceptive awareness and sensitivity in the eating disorders literature, the application of these principles in treatment has received minimal attention. Interoceptive exposure (IE) is a behavior therapy intervention, originally developed for the treatment of panic disorder, which aims to increase tolerance to the physical symptoms of anxiety through repeated provocation of personally relevant somatic sensation associated with feared outcomes. Given the exaggerated anxiety related to food and eating which contributes to food avoidance in AN (27), as well as the high rates of alexithymia (39), IE has begun to gain support as a transdiagnostic intervention strategy that can be integrated into cognitive–behavioral therapy oriented (CBT) ED treatment (47). This is on the bases of its ability to not only modify interoceptive sensitivity but also improve interoceptive clarity through identification and labeling of emotion (reviewed in Boswell et al.). While strategies such as exposure to hierarchies of “forbidden” foods, supervised meal exposures and body shape/mirror exposure are already well established in ED specific CBT (48), current literature suggests a more explicit integration of IE into these treatment strategies, which may be advantageous. Notably however, controlled trials published to date have failed to show the effectiveness of IE in clinical practice. An ongoing randomized control trial, predicted to be completed in 2022, is considering the use of implementing this approach pharmacologically in patients with AN by using infusions of isoproterenol to repeatedly trigger cardiorespiratory sensations and anxiety during meal anticipation (49). Clearly, further pilot studies and preliminary trials are needed to examine the process and impact of AN-specific IE techniques, which may allow the development of more effective adjunctive treatment methods.

## Discussion

### Findings

**Figure 2:**
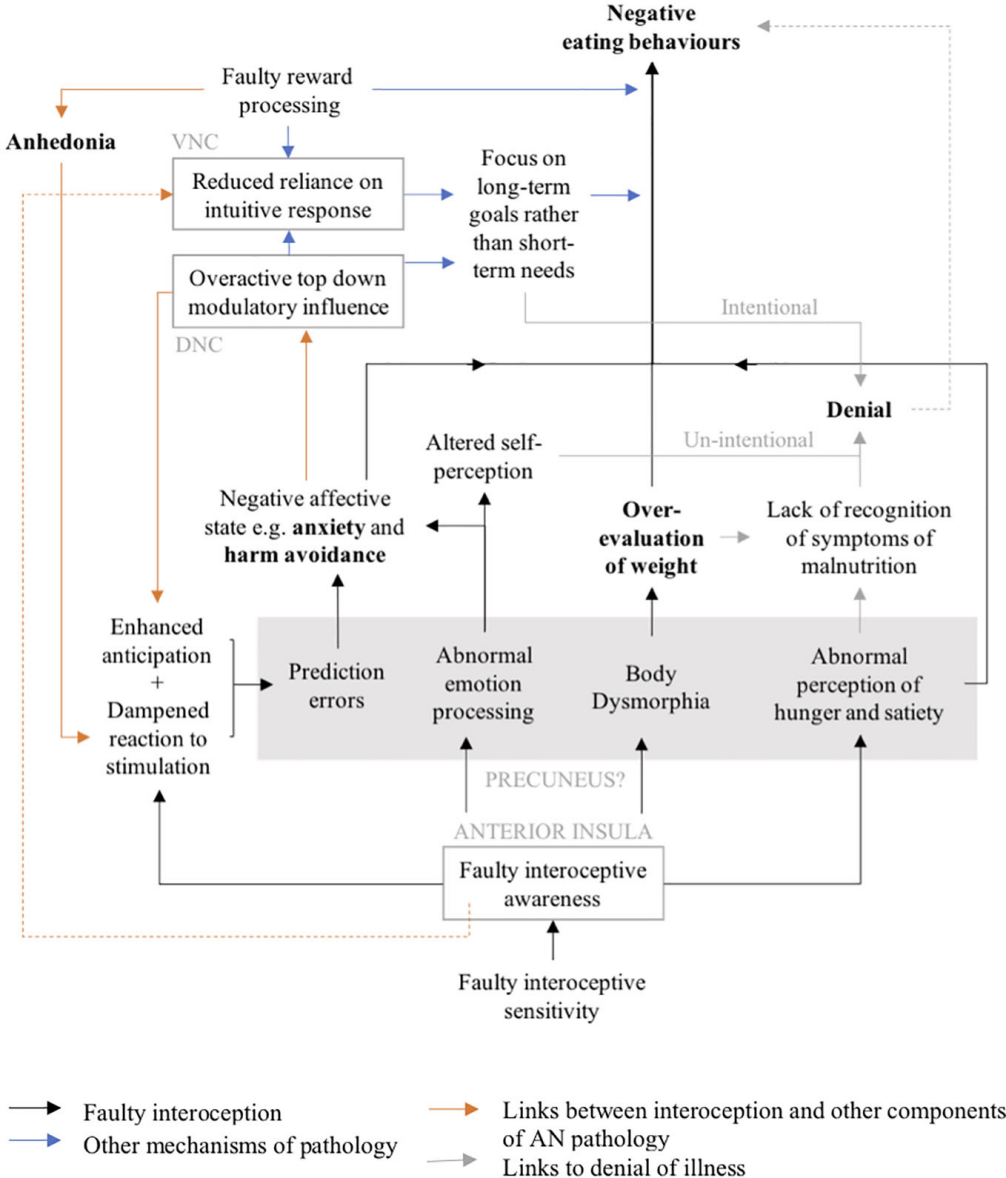
Model for the neuro-circuitry of AN: VNC, ventral (limbic) neuro-circuit—primarily ventral striatum; DNC, dorsal (cognitive) neuro-circuit—primarily dorsolateral prefrontal cortex.

While often under-represented, several lines of evidence suggest interoceptive abnormalities may play a role in the pathophysiology of AN. Specifically, their contributions to abnormal perception of hunger and satiety, body dysmorphia, abnormal emotion processing, and prediction errors have been summarized in **Figure 2**, which proposes a novel model for the neuro-circuitry of AN with interoception at its core.

### Clinical Implications

All four of these domains have been shown to contribute to both the symptomatology observed in AN as well as the barriers to treatment. The link between interoceptive deficits and body dysmorphia—leading to over-evaluation of weight and subsequent negative eating behaviors—could potentially be central to the pathology of AN, while altered self-perception due to abnormal emotion processing and perception of hunger and satiety is likely to contribute to lack of recognition, or ‘denial’ of the severity of starvation, which is one of the fundamental challenges in treating the disorder. It is important nonetheless that the potentially central role for interoception be considered in the context of broader literature on AN. **Figure 2** also summarizes the possible interactions between faulty interoception and other components of AN pathology. Importantly, the authors hypothesize that the role of abnormal emotion processing and prediction errors in creating negative affective states contributes to overactive top-down modulatory influences and subsequent focus on long-term goals rather than short term needs. This is compounded by well-established deficits in reward processing, leading to a reduced reliance on intuitive responses and thereby the perpetuation of negative eating behaviors and intentional denial of illness.

### Strengths and Limitations

Neuroimaging studies suggesting the anterior insula as a key multimodal integrative region (5, 7–10) with abnormal structure and function in AN (11, 27–29, 31, 34, 35, 46) further support a role for interoception deficits in the symptomatology of AN. The novel model of neuro-circuitry proposed in this review can be integrated comfortably with this existing literature, adding a further level of understanding to the neurobiological etiology of AN. Nonetheless, the hypothesis offered here does not constitute a systematic review and is hindered by a paucity of guiding literature. Specifically, prospective longitudinal studies looking at differentiating between genetically-determined trait alterations and state alterations secondary to malnutrition are challenging given the age group affected, incidence of AN, and length of follow-up necessary. Because of this, research has focused on recovered AN patients who are not affected by state related changes, thereby limiting the ability to draw conclusions about the acutely ill phase of AN. In addition, research is often confounded by common comorbid diagnoses including depression and anxiety as well as the inclusion of subjects at varying stages of illness or treatment due to the problematic definition of ‘recovery’ in AN.

### Conclusion and Future Research Directions

Future directions will undoubtedly include detailed studies of the neuroanatomical correlates of interoception in AN, aided by development of new methods to explore modalities of visceral sensitivity. In addition, ongoing research with interoception and its neurobiology at its core will allow a better understanding of how faulty interoception may contribute to illness denial in AN, which is currently one of the major obstacles to recovery. Given the importance of objective tests of interoception in future research, it is clear that the techniques currently used to measure interoceptive sensitivity (specifically HBTs) must be given due scrutiny, with every effort made to establish more reliable methods of investigating a key component of this devastating illness. Finally, further refinement of the novel model of AN pathology proposed in this review may support the integration of IE techniques into AN-specific CBT or development of more efficacious forms of treatment than those currently available.

## Author Contributions

AJ was responsible for the conception of the review as well as drafting all versions of the article. RP provided comments and critical revisions during the editing process and both authors were responsible for final approval of the version to be published.

## Conflict of Interest

The authors declare that the research was conducted in the absence of any commercial or financial relationships that could be construed as a potential conflict of interest.
